# Plant-mSubP: a computational framework for the prediction of single- and multi-target protein subcellular localization using integrated machine-learning approaches

**DOI:** 10.1093/aobpla/plz068

**Published:** 2019-10-17

**Authors:** Sitanshu S Sahu, Cristian D Loaiza, Rakesh Kaundal

**Affiliations:** 1 Department of Electronics and Communication Engineering, Birla Institute of Technology, Mesra, Ranchi, India; 2 Department of Plants, Soils, and Climate/Center for Integrated BioSystems, College of Agriculture and Applied Sciences, Utah State University, Logan, UT, USA; 3 Bioinformatics Facility, Center for Integrated BioSystems, Utah State University, Logan, UT, USA

**Keywords:** Artificial intelligence, machine learning, multi-location, prediction tool, protein science, subcellular localization, web server

## Abstract

The subcellular localization of proteins is very important for characterizing its function in a cell. Accurate prediction of the subcellular locations in computational paradigm has been an active area of interest. Most of the work has been focused on single localization prediction. Only few studies have discussed the multi-target localization, but have not achieved good accuracy so far; in plant sciences, very limited work has been done. Here we report the development of a novel tool Plant-mSubP, which is based on integrated machine learning approaches to efficiently predict the subcellular localizations in plant proteomes. The proposed approach predicts with high accuracy 11 single localizations and three dual locations of plant cell. Several hybrid features based on composition and physicochemical properties of a protein such as amino acid composition, pseudo amino acid composition, auto-correlation descriptors, quasi-sequence-order descriptors and hybrid features are used to represent the protein. The performance of the proposed method has been assessed through a training set as well as an independent test set. Using the hybrid feature of the pseudo amino acid composition, N-Center-C terminal amino acid composition and the dipeptide composition (PseAAC-NCC-DIPEP), an overall accuracy of 81.97 %, 84.75 % and 87.88 % is achieved on the training data set of proteins containing the single-label, single- and dual-label combined, and dual-label proteins, respectively. When tested on the independent data, an accuracy of 64.36 %, 64.84 % and 81.08 % is achieved on the single-label, single- and dual-label, and dual-label proteins, respectively. The prediction models have been implemented on a web server available at http://bioinfo.usu.edu/Plant-mSubP/. The results indicate that the proposed approach is comparable to the existing methods in single localization prediction and outperforms all other existing tools when compared for dual-label proteins. The prediction tool will be a useful resource for better annotation of various plant proteomes.

## Teaser

In order for various cellular processes to be carried out in a cell, the correct identification of subcellular localization of a protein is crucial. Determining the localization of proteins through experiments can be laborious, expensive and time-consuming. Computational prediction is thus essential to decipher protein function and faster genome annotation. It also aids the identification of drug targets. We present here a software, Plant-mSubP, which is a publicly available web platform at http://bioinfo.usu.edu/Plant-mSubP/. Plant-mSubP uses artificial intelligence to effectively predict the protein localizations (11 single- and three dual-target) inside the plant cell. This resource can assess a high number of proteins in order to find their desired location in a cell, and thus will be useful in better annotation of various plant proteomes.

## Background

The cell is a three-dimensional space composed of several compartments, having different physicochemical environment and function. For efficient functioning, the cell’s functional machinery - protein needs to be present at specific cellular compartments. Improper localization of proteins may result in disease and cell death (Park *et al.* 2011; Mer and Andrade-Navarro 2013). Therefore, subcellular location is an essential attribute in the functional characterization of proteins (Casadio *et al.* 2008). In recent years, knowledge about protein subcellular localization has earned enormous attention due to its important roles in elucidating protein functions, identifying drug targets and many more (Chou and Cai 2005). Thus, predicting the subcellular localization of protein is an important issue in proteomics. Since biochemical experiments are expensive and time-consuming, computational approaches gained an attention in prediction of subcellular localization. Several *in silico* approaches have been proposed to predict the subcellular localization. In one of the approach as reported in Lin *et al.* (2011) the motifs recognized by the sorting proteins and receptors of the protein transport machinery to move protein products from the cytosol to other subcellular localizations. This method is limited by the knowledge of sorting signals and absence of known motifs. In Adelfio *et al.* (2013), they used the sequence homology feature to proteins of experimentally verified localizations with the assumption that similar proteins target similar localizations. There are many deviations of this rule which may mislead the prediction (e.g. the proteins of the Lsg1 family of GTPases). Further, in some other methods, it uses protein sequence features such as amino acid composition, dipeptide composition, pseudo amino acid composition based on the assumption that the physicochemical properties of the protein residues may somehow be coupled to the physicochemical properties of the environment where the protein performs its function. Therefore, the differences in environments will be engraved in the protein amino acid compositions (Nakashima and Nishikawa 1994; Nielsen *et al.* 1997; Emanuelsson *et al.* 2000; Chou 2001; Höglund *et al.* 2006; Mak *et al.* 2008). The advantage of this approach is that it can be applied to any set of compartments and proteins, provided there is enough availability of data. Several approaches have been developed on annotation-based methods. Recently, Gene Ontology (GO)-based features have gained popularity for the prediction of protein subcellular locations (Chou and Cai 2004; Wan *et al.* 2011, 2012a, b, 2013; Mei 2012). Also, a combination of GO, composition and evolutionary features have been successfully used. To date, the GO-based features have shown better accuracy in predicting the subcellular localizations in both single- and multi-label localizations (Chou and Shen 2006). Although it shows superior results, it has several bottlenecks.

The set of distinct GO terms derived from a given data set may not be representative for other data sets; means the generalization capabilities of the predictors may be weakened when new GO terms outside the predefined GO term set are found in the test proteins. The GO term s*et al*so varies from species to species. Although the GO-based model looks promising, there are no specific classes defined for the multi-located proteins. Since overall actual accuracy is the most desired measure in multi-located classes, the existing GO-based models do not show up the actual accuracy of the multi-class proteins which is misleading the accuracy performance. In addition to this, in the existing multi-target approaches, there have been no report of comparing the performances of different data sets, e.g. how the models developed from single-label proteins differ from the models developed on a combined set of single- and multi-target proteins data set, or the models developed from multi-target protein data sets only.

Most of the existing methods are limited to the prediction of single-location proteins. These methods generally exclude the occurrences of multi-label proteins. But the fact is, multi-location proteins exist that can simultaneously reside at, or move between, two or more different subcellular localizations (Chou and Shen 2007; Chou *et al.* 2011; Lin *et al.* 2011; Xiao *et al.* 2011; Wu *et al.* 2012). Recently, several multi-label predictors such as Plant-mPLoc (Chou and Shen 2010a), Virus-mPLoc (Shen and Chou 2010), iLoc-Plant (Wu *et al.* 2011), iLoc-Virus (Xiao *et al.* 2011), HybridGO-Loc (Wan *et al.* 2014), Y-Loc (Briesemeister *et al.* 2010) and mGOASVM (Wan *et al*. 2012a) have been proposed. These predictors use the GO information and have demonstrated superiority over existing methods. Some other methods are based on predicting the transit peptides; Sperschneider *et al.* (2017) proposed a web tool, LOCALIZER for predicting plant and effector protein localization to three classes; chloroplast, mitochondria and nuclei. Chen *et al.* (2017) proposed a method to identify the peroxisome subcellular locations in plants. BUSCA (Savojardo *et al.* 2018) combines different computational tools to predict signal and transit peptides, GPI anchors and transmembrane domains. It has one module available for plant proteins but no option for predicting dual- or multi-label proteins.

Many subcellular predictors have been developed especially for specific species (Kaundal and Raghava 2009; Kaundal *et al.* 2013). A different promising approach has been proposed based on account amino acid composition at different levels of amino acid exposure (Emanuelsson *et al.* 2000). Efficient feature representation of a protein is a very important aspect of subcellular localization (Chou and Shen 2007). Hence there is a demand to accurately predict the subcellular localizations efficiently which further helps in the correct annotation of various proteomes.

In literature, dual targeting of a multitude of proteins has been described for native plant proteins (Peeters and Small 2001; Silva-Filho 2003; Mackenzie 2005; Mitschke *et al.* 2009). Also, protein folding, post-translational modification and protein–protein interactions can be involved in determining the targeting of proteins with multiple sites of action (Karniely and Pines 2005; Mitschke *et al.* 2009). It has been seen that various amino acid features significantly contribute to the dual targeting of localizations (Mitschke *et al.* 2009).

In this paper, we propose a simple and efficient predictor tool based on the sequence features. It can be used to classify single- and dual-label proteins subcellular localization. The system predicts the 11 single localizations (cell membrane, cell wall, plastid, cytoplasm, endoplasmic reticulum, extracellular, Golgi apparatus, mitochondrion, nucleus, peroxisome and vacuole) and three dual-localized protein classes (cytoplasm-nucleus, mitochondrion-plastid and cytoplasm-Golgi apparatus). Various sequence-based features of a protein sequence viz. amino acid composition (AAC), dipeptide composition (DIPEP), pseudo amino acid composition (PseAAC), N-_terminal_-Center-C-_terminal_ (NCC) composition, physicochemical properties, Composition and Transition, and Quasi-sequence-order-based methods, and a range of hybrid features are explored in a machine learning framework to develop diverse prediction models for better confidence and reliability.

### Implementation

For the development of any useful sequence-based statistical predictor for a biological system as reported in a series of recent publications (Chou *et al.* 2011; Wu *et al.* 2011; Lin *et al.* 2013; Chen *et al.* 2018; Feng *et al.* 2019), one should implement the 5-step rules (Chou *et al.* 2011) such as (i) construction of a valid benchmark data set to train and test the predictor; (ii) mathematical representation of biological sequence samples which will reflect their intrinsic correlation with the target to be predicted; (iii) an algorithm (or engine) for performing prediction operation; (iv) cross-validation tests to objectively evaluate the anticipated accuracy of the predictor; and (v) a user-friendly web server/tool for the predictor that is easily accessible to the public. We have implemented our best-performing prediction models on the publicly available tool, Plant-mSubP and is freely accessible on the web.

### Data sets generation

To develop an efficient prediction system, it is important to first gather data sets of known subcellular localization and extract diverse relevant features out of it for use in the training and testing of machine learning classifiers. The protein sequences of all the plants were extracted from the UniProt database release 2018_02 (http://www.uniprot.org) using [keywords: SUBCELLULAR LOCATION AND reviewed: yes]. Sequence annotations marked as ‘PROBABLE’, ‘POSSIBLE’ and ‘BY SIMILARITY’ were discarded. This resulted in 16 494 unique sequences of proteins, annotated to 14 different single- and dual-label subcellular localizations as detailed in Table 1.

**Table 1.** Distribution of subcellular localization classes (single- and dual-located) for all plant data from UniProt database release 2018_02 in the training data set and independent testing data set. *About 10 % of sequences from the original training data set were kept separate for independent testing. In total, 16 494 plant protein sequences were found after applying the filters [viridiplantae AND annotation:(type: location confidence: experimental)].

Type	Subcellular location	# sequences retrieved	# sequences after redundancy check (30 % cut-off)	*Training data set	Training data set (sequences length > 50)	Independent data set (sequences length > 50)
Single label	Plastid	11 302	2979	2678	2468	248
	Cytoplasm	739	403	361	351	40
	Extracellular	237	186	166	140	14
	Nucleus	734	636	571	568	63
	Mitochondrion	759	537	481	447	52
	Cell membrane	1256	927	830	829	92
	Golgi apparatus	277	229	204	204	23
	Endoplasmic reticulum	393	320	285	280	29
	Vacuole	260	198	176	176	20
	Peroxisome	80	63	57	57	06
	Cell wall	52	47	42	37	05
Dual label	Mito-plastid	141	133	118	118	13
	Cyto-nucleus	210	196	175	170	20
	Cyto-Golgi	54	38	34	34	04
	Total	16 494	6892	6178	5879	629

After reducing the sequence identity with a cut-off of <30 % using BlastClust, a total of 6892 proteins were left for further use. This was done within the class as well as across the classes. About 10 % of these data, i.e. 714 sequences, were kept separate for independent testing; thus, a total of remaining 6178 proteins constituted our initial training data set (column 5, Table 1). Testing on independent data sets that are not used during the machine learning model development has been reported to be the best benchmark to test the performance of various prediction modules. Further, to remove any potential fragments, 5879 sequences were extracted out of the 6178 proteins by filtering those sequences whose length was greater than 50 (column 6, Table 1) and were used in the training/testing of various machine learning algorithms. Similarly, in the independent test data, out of 714 proteins, 629 sequences were extracted whose length was greater than 50 (column 7, Table 1).

### Feature representation methods

With the explosive growth of biological sequences, one of the most important and difficult problems in computational biology is the expression of a biological sequence with a discrete model or a vector, yet retaining sequence-order information or key pattern characteristics. In this work, the following diverse features have been used:

1. Amino acid composition (AAC)
2. Dipeptide composition (DIPEP)
3. Pseudo amino acid composition (PseAAC)
4. Terminal-based N-Center-C (NCC) amino acid composition

The above four features have been explained in detail in our previous studies on the identification and characterization of various plastid types (Kaundal *et al.* 2013), and so not discussed here. In the current study, we wanted to explore these features to see if they could predict the multi-target protein localizations as well. In addition, we extracted and implemented the following diverse features from protein sequences to achieve high accuracy.

1. Physicochemical property-based composition

The physicochemical properties of amino acids are successfully used for prediction of protein function, structure and subcellular localizations with various alterations. In literature, it has been shown that the physicochemical properties such as acidic, basic, hydrophobicity, hydrophilicity, neutral and atomic composition play an important role in the residing the protein the cellular compartment. Compositions of amino acids of these classes are calculated as a feature to represent the protein. Thus, each protein is represented by a 26-dimensional feature vector.

1. Composition and Transition

A protein sequence can be represented and categorized into three classes according to its attributes (Dubchak *et al.* 1999), where each amino acid in the sequence is encoded as 1, 2 or 3 depending on the class that it belongs to. The attributes used here are hydrophobicity, normalized van der Waals volume, polarity and polarizability. The corresponding classification for each attribute is listed in Table 2.

**Table 2.** Group attributes and classification of various amino acids in a protein, as defined in Dubchak *et al.* (1999).

	Group 1	Group 2	Group 3
Hydrophobicity	Polar	Neutral	Hydrophobicity
	R, K, E, D, Q, N	G, A, S, T, P, H, Y	C, L, V, I, M, F, W
Normalized van der Waals volume	0–2.78	2.95–4.0	4.03–8.08
	G, A, S, T, P, D, C	N, V, E, Q, I, L	M, H, K, F, R, Y, W
Polarity	4.9–6.2	8.0–9.2	10.4–13.0
	L, I, F, W, C, M, V, Y	P, A, T, G, S	H, Q, R, K, N, E, D
Polarizability	0–1.08	0.128–0.186	0.219–0.409
	G, A, S, D, T	C, P, N, V, E, Q, I, L	K, M, H, F, R, Y, W
Charge	Positive	Neutral	Negative
	K, R	A, N, C, Q, G, H, I, L, M, F, P, S, T, W, Y, V	D, E
Secondary structure	Helix	Strand	Coil
	E, A, L, M, Q, K, R, H	V, I, Y, C, W, F, T	G, N, P, S, D
Solvent accessibility	Buried	Exposed	Intermediate
	A, L, F, C, G, I, V, W	R, K, Q, E, N, D	M, S, P, T, H, Y

After this classification, three types of descriptors: composition (C), Transition (T) and Distribution (D) are calculated.

1. Composition (CTDC)

The composition is defined as the global percentage for each of the encoded classes in a protein sequence.

$$\begin{array}{l} \begin{array}{ll} C_{r}=\frac{n_{r}}{N} & r=1,2,3 \end{array} \end{array}$$ (1)

where *n*_*r*_ is the number of amino acids of type *r* in the encoded sequence; *N* is the length of the sequence.

1. Transition (CTDT)

Transition is defined as each of the changes between classes for the encoded sequences, a transition from class 1 to 2 is the percent frequency with which 1 is followed by 2 or 2 is followed by 1 in the encoded sequences.

$$\begin{array}{l} \begin{array}{ll} T_{rs}=\frac{n_{rs}-n_{sr}}{N-1} & r=12,13,23 \end{array}\;\;\; \end{array}$$ (2)

where *n*_*rs*_, *n*_*sr*_ are the numbers of dipeptide encoded as *rs* and *sr* in the sequence; *N* is the length of the sequence.

1. Quasi-sequence-order descriptors (QSO)

The QSO descriptors are derived from the distance matrix between the 20 amino acids. Based on the definitions and figures used in protr package (Xiao *et al.* 2015) for the equations originally described in Chou (2000), a quasi-sequence-order descriptor can be defined for each of the amino acids as:

$$\begin{array}{l} \begin{array}{ll} X_{r}=\frac{f_{r}}{\overset{20}{\underset{r=1}{\sum }}f_{r}+w\overset{maxlag}{\underset{d=1}{\sum }}\tau_{d}} & r=1,2,\ldots ,20 \end{array} \end{array}$$ (3)

where *f*_*r*_ is the normalized occurrence for amino acid type *i* and *N* is a weighting factor (*w* = 0.1). These are the first 20 quasi-sequence-order descriptors. The other 30 quasi-sequence-order are defined as:

$$\begin{array}{l} \begin{array}{ll} X_{d}=\frac{w\tau_{d-20}}{\overset{20}{\underset{r=1}{\sum }}f_{r}+w\overset{maxlag}{\underset{d=1}{\sum }}\tau_{d}} & r=21,22,\ldots ,maxlag \end{array}\;\;\; \end{array}$$

### Support Vector Machines

Support Vector Machine (SVM) is a machine learning technique first introduced by Cortes and Vapnik (1995). It is a statistical learning theory based on optimization principle. This technique has been used in the field of image processing, speech processing, protein subcellular localization prediction, protein secondary structure prediction and many other areas. The main aim of SVM is to separate the training data by maximization the margin with maximum computing efficiency. Multi-class classification is implemented by using a series of binary classification. Many methods have been used for multi-class classification like Directed Acyclic Graph Support Vector Machines (DAGSVM), One-vs.-Rest (OvR) and One-vs.-One (OvO). Radial basis function (RBF) is a popular kernel widely used for classification. In our study, we have used OvR strategy which involves training a single classifier per localization class, with the samples of that class as positive samples and all other localization classes as negatives. Making decisions means then applying all classifiers to an unseen sample and predicting the label for which the corresponding classifier reports the highest confidence score. The idea here is to reduce the problem of multi-class classification to multiple binary classification problems.

#### Training/testing schema.

We have used 5-fold cross-validation technique for training/testing procedure, using the OvR strategy for decision-making. Here, the training data are divided into five parts. For development of model, four parts are combined to form a training set and fifth part is used as testing data set. This process is repeated five times by changing the training and testing data set. Finally, the models are tested on an independent data set called as validation set.

#### Evaluation parameters.

The evaluation of models is done based on following parameters.

(i) Sensitivity: It is defined as a percent of truly predicted true proteins,

$$\begin{array}{l} Sensitivity\;(S_{n})=\frac{TP}{TP+FN} \end{array}$$ (4)

1. Specificity: It is the percent of non-true correctly predicted as non-true proteins,

$$\begin{array}{l} Specif\;icity\;(SP)\;=\frac{TN}{TN+FP}\;\;\;\times \;\;\;100 \end{array}$$ (5)

1. Accuracy: It is the percentage of correctly predicted proteins (true and non-true proteins),

$$\begin{array}{l} Accuracy\;(ACC)\;=\frac{TP+TN}{TP+FN+FP+FN}\times 100 \end{array}$$ (6)

1. Precision: It is the percentage of positive predictions those are correct calculated as:

$$\begin{array}{l} Precision=\frac{TP}{TP+FP}\;\;\;\times \;\;\;100 \end{array}$$ (7)

(v) Rate of False Predictions (RFP): It is defined as the probability of false predictions percentage from the predictions set,

$$\begin{array}{l} RFP=\frac{FP}{TP+FP}\;\;\;\times \;\;\;100 \end{array}$$ (8)

1. Error Rate (ER): ER is defined as the percentage of misclassified samples,

$$\begin{array}{l} (ER)=\frac{FP+FN}{TP+FN+FP+TN}\;\;\;\times \;\;\;100 \end{array}$$ (9)

1. Matthews Correlation Coefficient (MCC): MCC is defined as the parameter for prediction of class. For perfect prediction, it is equal to 1 and 0 for random prediction. It is given by

$$\begin{array}{l} MCC\;=\frac{\left(TP\times TN)-(FP\times FN\right)}{\sqrt{\left(TP+FP)(TP+FN)(TN+FP)(TN+FN\right)}} \end{array}$$ (10)

where TP = True Positives, TN = True Negatives, FP = False Positives, and FN = False Negatives.

## Results and Discussion

**Figure 1.**
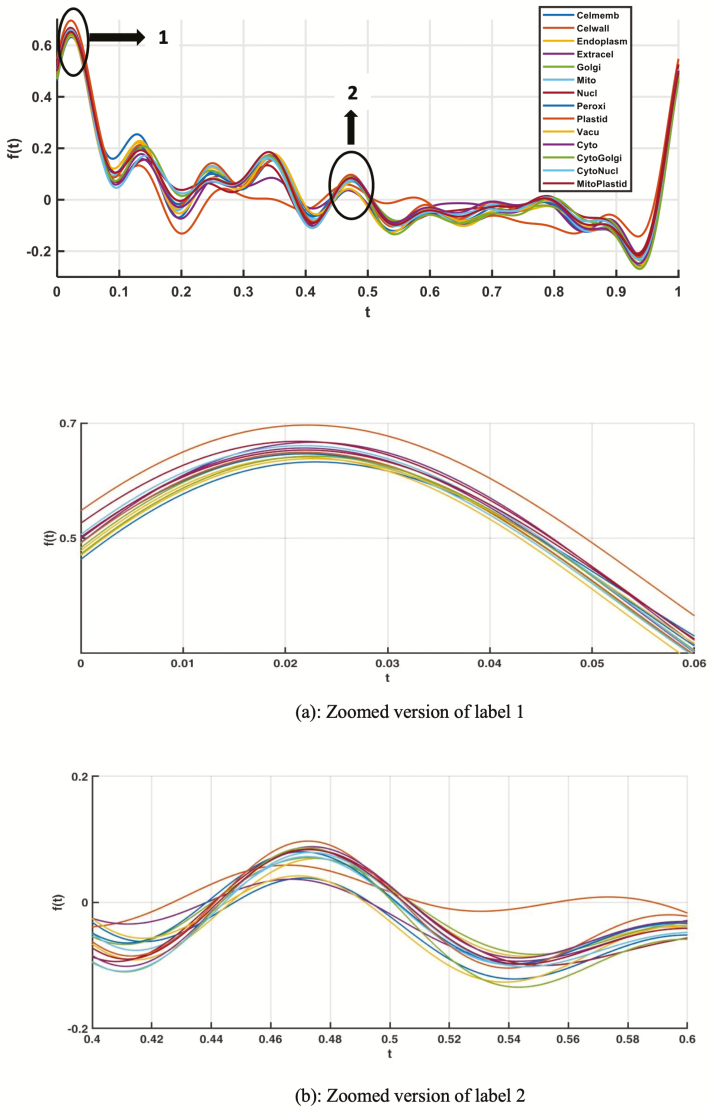
Andrews plot of amino acid composition (AAC) feature for all the single- and dual-label localizations.

**Figure 2.**
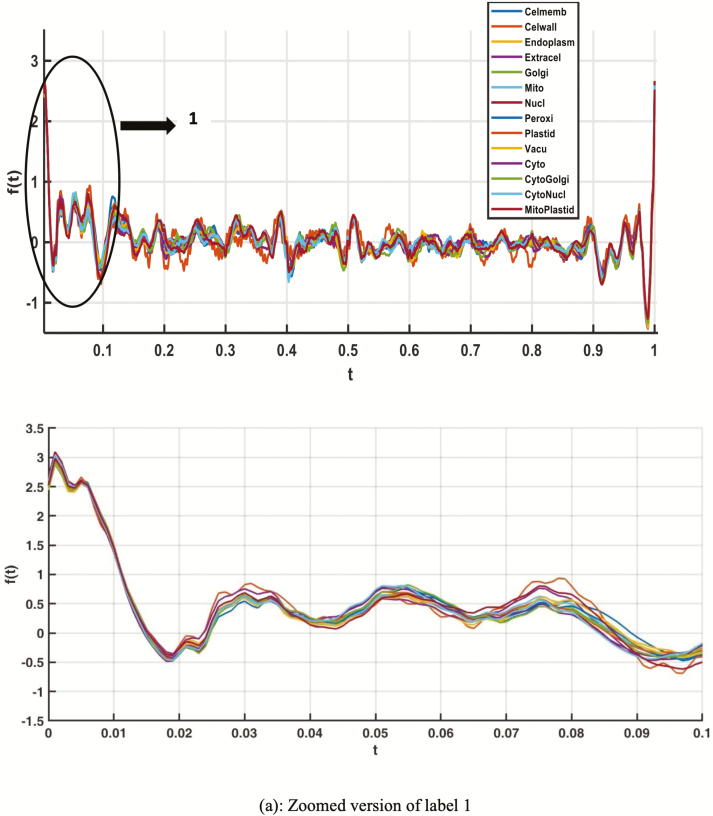
Andrews plot of PseAAC-NCC-DIPEP feature for all the single- and dual-label localizations.

To assess the distinguishing capability of various protein features, we first studied the Andrews plot. Andrews plot is a method in high dimensional data to visualize the latent structure. It has been used to represent multivariate data. The Andrews plot of amino acid composition (AAC) and the PseAAC-NCC-DIPEP features is shown in Figs 1 and 2.

From the variations in the plots, it can be elucidated that the extracted features are capable to distinguish the different localization classes. This shows that composition-based models and other sequence features could be used in a machine learning framework to develop prediction models for classifying protein sequences of different subcellular localizations.

### Five-fold cross-validation training/testing

In this study, the 5-fold cross-validation technique was used with SVM as the prediction model. The performance of various models was evaluated based on various statistical parameters as explained above. In a 5-fold cross-validation test in the training data set, the overall accuracy of the SVM model results is listed in Table 3. It shows that the PseACC-NCC-DIPEP model provides the superior result on all three types of data sets; single-label, single- and dual-label combined, and dual-label proteins. The PseAAC-NCC-DIPEP feature achieves an overall accuracy of 81.97 % on the single-label training set (Table 3a), 84.75 % on the single- and dual-label combined training data set (Table 3b) and 87.88 % on the dual-label only proteins data set (Table 3c). We did not see a significant difference in prediction performances across the data set types as depicted in Table 3a–c; for example, in the PseACC-NCC-DIPEP model, there is a marginal increase of 3.3 % accuracy of the dual-label model over the combined data set module. It is worth mentioning here that in our separate comparative analysis (results not shown), we also explored the use of Artificial Neural Networks (ANN) but achieved much lower overall accuracies in a 5-fold training/testing procedure as compared to the SVMs.

**Table 3.** (a) Performance comparison by 5-fold cross-validation testing on the training data set of single-label proteins using SVMs; (b) Performance comparison of 5-fold cross-validation testing on the combined training data set (single- + dual-label) using SVMs; (c) Performance comparison of 5-fold cross-validation testing on the dual-localized training data set using SVMs. Bold values represents the best performance. RBF = radial basis function of SVM; C = regularization parameter.

(a)				
Feature representation methods	Overall accuracy (%) (single-label data)			
AAC (σ = 2, C = 10)	73.65			
DIPEP (σ = 50, C = 500)	77.56			
PseAAC (σ = 10, C = 500)	75.49			
NCC (σ = 10, C = 50)	74.36			
**PseAAC-NCC-DIPEP** (σ = 50, C = 500)	**81.97**			
NCC-DIPEP (σ = 50, C = 500)	81.18			
QSO (σ = 10, C = 500)	73.25			
NCC-DIPEP-CTDC-CTDT- QSO (σ = 5, C = 30)	80.42			
(b)				
Feature representation methods	Overall accuracy (%) (single- + dual-label data)			
AAC (σ = 2, C = 10)	68.48			
DIPEP (σ = 50, C = 500)	74.59			
PseAAC (σ = 10, C = 500)	71.87			
NCC (σ = 10, C = 50)	70.74			
**PseAAC-NCC-DIPEP** (σ = 50, C = 500)	**84.75**			
NCC-DIPEP (σ = 50, C = 500)	83.96			
Physicochem [atomi + hydrophobicity, basic]	73.21			
NCC-DIPEP-physicochem	83.79			
Quasi-sequence-order descriptors	54.38			
NCC-DIPEP-CTDC-CTDT- QSO	60.02			
(c)				
Model	Kernel	C	Gamma	Overall accuracy (%) (dual- label data)
AAC	RBF	10	0.001	76.64
DIPEP	RBF	10	0.001	82.29
PseAAC	RBF	10	0.001	77.63
NCC	RBF	10	0.001	86.02
NCC-DIPEP	RBF	10	0.001	87.57
**PseAAC-NCC-DIPEP**	RBF	10	0.001	**87.88**

#### Independent testing/benchmarking.

Next, we performed a test on the independent data sets, the 10 % data that were kept separate for testing (as in Table 1). The comparison results are reported in Table 4. As reported in previous studies (Chou and Elrod 1999; Kaundal and Raghava 2009; Kaundal *et al.* 2010, 2013; Tung *et al.* 2017), the best way to test the prediction performance of a particular tool is to test it on independent data sets, which have not been used in the process of training/testing of machine learning. From the results in Table 4a–c, it shows that the PseAAC-NCC-DIPEP feature is superior providing an overall accuracy of 64.36 % on the single-label data set, 64.84 % on the single- and dual-target combined data set and 81.08 % accuracy on the independent dual-target data set. This shows that the dual-target proteins might contain some specialized signals for dual targeting which are not well represented when we develop training classifiers on a combined data. The overall results show that PseAAC-NCC-DIPEP feature is superior in predicting the single- and dual-label subcellular localizations.

**Table 4.** (a) Comparison of prediction results on an ‘independent data set’ based on models trained from single-label proteins using SVMs; (b) Comparison of prediction results on an ‘independent data set’ based on models trained from combined data set (single- + dual-label); (c) Comparison of prediction results on an ‘independent data set’ based on models trained from dual-label proteins data set. Bold values represents the best performance.

(a)				
Feature representation methods	Accuracy (%)			
AAC (σ = 2, C = 10)	59.11			
DIPEP (σ = 50, C = 500)	59.11			
PseAAC (σ = 10, C = 500)	59.12			
NCC (σ = 10, C = 50)	50.34			
**PseAAC-NCC-DIPEP** (σ = 50, C = 500)	**64.36**			
NCC-DIPEP (σ = 50, C = 500)	64.05			
QSO (σ = 10, C = 500)	57.05			
NCC-DIPEP-CTDC-CTDT-QSO (σ = 5, C = 300)	61.46			
(b)				
Feature representation methods	Accuracy (%)			
AAC (σ = 2, C = 10)	57.71			
DIPEP (σ = 50, C = 500)	58.95			
PseAAC (σ = 10, C = 500)	56.60			
NCC (σ = 10, C = 50)	52.88			
**PseAAC-NCC-DIPEP** (σ = 50, C = 500)	**64.84**			
NCC-DIPEP (σ = 50, C = 500)	64.42			
Quasi-sequence-order descriptors	58.94			
NCC-DIPEP-CTDC-CTDT-QSO	38.49			
(c)				
Model	Kernel	C	Gamma	Accuracy (%)
AAC	RBF	10	0.001	72.56
DIPEP	RBF	10	0.001	72.97
PseAAC	RBF	10	0.001	75.67
NCC	RBF	10	0.001	78.37
NCC-DIPEP	RBF	10	0.001	75.67
**PseAAC-NCC-DIPEP**	RBF	10	0.001	**81.08**

#### Comparison with other existing tools.

Further, we assessed the performance of our tool, Plant-mSubP with the existing tools for predicting both the single- and dual-label subcellular localizations. In literature, many methods have been reported to predict the subcellular localizations but most of them are for single-class proteins. In this paper, we have compared our method with the existing methods for plant subcellular localization such as YLoc (Briesemeister *et al.* 2010), Euk-mPloc (Chou and Shen 2010b) and iLoc-Plant (Wu *et al.* 2011) that were developed for multi-label proteins. The prediction results for the YLoc, Euk-mPloc and iLoc-Plant are assessed on the independent data set as created in Table 1. The comparison results are reported in Table 5. The results show that our proposed method is better than the three compared tools to predict the subcellular localizations, single- as well as dual-target proteins. We believe Plant-mSubP will be helpful in better annotation of the existing and novel plant proteomes.

**Table 5.** Comparison of actual prediction accuracy of Plant-mSubP on an ‘independent data set’ with the existing web tools that support multi-label localizations. Actual accuracy is calculated (in percentage) as the ratio of number of localization samples correctly predicted divided by the total number of samples in the independent data set.

Web tools	Prediction accuracy (%) (single- + dual-label data)	Prediction accuracy (%) (dual-label data)
YLoc	34.35	35.89
Euk-mPloc 2.0	53.5	44.86
iLoc-Plant	37.42	34.42
Our method [Plant-mSubP]	64.84	81.08

### Tool development and availability

In various recent publications (Chou and Shen 2009; Kaundal and Raghava 2009; Kaundal *et al.* 2010, 2013; Chou 2011, 2013; Chou *et al.* 2011; Chen *et al.* 2018; Feng *et al.* 2019), it is demonstrated that user-friendly and openly accessible web tools represent the future direction for developing practically more useful computational tools.

From our analysis, the best-performing prediction algorithms were implemented on the web server called, Plant-mSubP (http://bioinfo.usu.edu/Plant-mSubP/). Its framework has been implemented using R, with the user interface and web server designed with the Shiny package. It has an intuitive interface in which the user can either upload a multi-FASTA format file or paste their sequences in a box. When the user submits a job, it will test the sanity of the sequences using protr R package; besides, it will check that the input sequences have a length more than 50 amino acids required to calculate N-Center-C terminal Composition features (Kaundal and Raghava 2009; Kaundal *et al.* 2010, 2013). The protein features extraction for Composition, Transition and Quasi-sequence-order descriptors are done using protr R package. Other features extraction was made with our in-house scripts in R. The web server currently supports a prediction workload up to a thousand sequences (1000).

Predictions methods implemented on the server were selected based on efficiency and fast-paced, including two options for a faster prediction (amino acid composition-based and dipeptide composition-based), two options for an accurate prediction using comprehensive hybrid features models (PseAAC-NCC-DIPEP and NCC-DIPEP-CTDC-CTDT-QSO) and a homology-search-based option (blastp). Support Vector Machines predictions were implemented using the e1071 R package. After the job submission, users can search throughout the results presented in an enriched table format or download a file with that information to be opened in a spreadsheet software (e.g. Excel); downloading the sequences alignments is also an option in case the user selects the homology-based BLAST approach for comparing the subcellular localization predictions with the machine-learned classifiers.

On the Plant-mSubP web server, we have also provided the links to download the sequences used to construct the predictions models (training sets) and the testing sequences used for independent test, separated by each subcellular localization class.

## Conclusion

An accurate prediction of protein localization is a very critical step in any functional genome annotation process. Various experimental procedures such as large-scale phenotyping screens, microarray or RNA-Seq experiments, protein–protein interaction assays etc. all rely heavily on the subcellular localization information. It is, therefore, necessary to continuously expand our knowledge in this area and develop highly accurate prediction tools. Although some tools exist to predict single localization of the proteins, very few have been developed for dual-targeting proteins and have limited accuracy. Very limited work has been reported for plant proteins. In this paper, we have developed an integrated machine learning framework to accurately predict the subcellular localizations of protein targeting for both the single and dual locations in plants. Various features of proteins have been explored and found that the PseAAC-NCC-DIPEP feature is superior in predicting the subcellular localizations for both single- and dual-targeting proteins. Using an independent data set for each localization class, we have compared our method with the available sequence-based prediction tools that also support dual-location prediction and found that our method, Plant-mSubP outperforms the existing methods. We believe the web server should be helpful to the users in the correct annotation of various proteomes.

## Availability and Requirements

Project name: Plant-mSubP
Project home page: http://bioinfo.usu.edu/Plant-mSubP/
Operating system(s): Linux
Programming language: R, Python, MATLAB
Other requirements: N/A
License: N/A
Any restrictions to use by non-academics: No restrictions to use this web tool
